# Molecular features of luminal breast cancer defined through spatial and single‐cell transcriptomics

**DOI:** 10.1002/ctm2.1548

**Published:** 2024-01-28

**Authors:** Ryohei Yoshitake, Hitomi Mori, Desiree Ha, Xiwei Wu, Jinhui Wang, Xiaoqiang Wang, Kohei Saeki, Gregory Chang, Hyun Jeong Shim, Yin Chan, Shiuan Chen

**Affiliations:** ^1^ Department of Cancer Biology and Molecular Medicine Beckman Research Institute of City of Hope Duarte California USA; ^2^ Department of Surgery and Oncology Graduate School of Medicine, Kyushu University Fukuoka Japan; ^3^ Integrative Genomics Core Beckman Research Institute of City of Hope Monrovia California USA; ^4^ Faculty of Veterinary Medicine Okayama University of Science Imabari Ehime Japan

**Keywords:** breast cancer, oestrogen receptor, intratumour heterogeneity, single‐cell RNA‐sequencing, spatial transcriptomics

## Abstract

**Background:**

Intratumour heterogeneity is a hallmark of most solid tumours, including breast cancers. We applied spatial transcriptomics and single‐cell RNA‐sequencing on patient‐derived xenografts (PDXs) to profile spatially resolved cell populations within oestrogen receptor‐positive (ER^+^) breast cancer and to elucidate their importance in oestrogen‐dependent tumour growth.

**Methods:**

Two PDXs of ‘ER‐high’ breast cancers with opposite oestrogen‐mediated growth responses were investigated: oestrogen‐suppressed GS3 (80–100% ER) and oestrogen‐dependent SC31 (40–90% ER) models. The observation was validated via single‐cell analyses on an ‘ER‐low’ PDX, GS1 (5% ER). The results from our spatial and single‐cell analyses were further supported by a public ER^+^ breast cancer single‐cell dataset and protein‐based dual immunohistochemistry (IHC) of SC31 examining important luminal cancer markers (i.e., ER, progesterone receptor and Ki67). The translational implication of our findings was assessed by clinical outcome analyses on publicly available cohorts.

**Results:**

Our space‐gene‐function study revealed four spatially distinct compartments within ER^+^ breast cancers. These compartments showed functional diversity (oestrogen‐responsive, proliferative, hypoxia‐induced and inflammation‐related). The ‘proliferative’ population, rather than the ‘oestrogen‐responsive’ compartment, was crucial for oestrogen‐dependent tumour growth, leading to the acquisition of luminal B‐like features. The cells expressing typical oestrogen‐responsive genes like *PGR* were not directly linked to oestrogen‐dependent proliferation. Dual IHC analyses demonstrated the distinct contribution of the Ki67^+^ proliferative cells toward oestrogen‐mediated growth and their response to a CDK4/6 inhibitor. The gene signatures derived from the proliferative, hypoxia‐induced and inflammation‐related compartments were significantly correlated with worse clinical outcomes, while patients with the oestrogen‐responsive signature showed better prognoses, suggesting that this compartment would not be directly associated with oestrogen‐dependent tumour progression.

**Conclusions:**

Our study identified the gene signature in our ‘proliferative’ compartment as an important determinant of luminal cancer subtypes. This ‘proliferative’ cell population is a causative feature of luminal B breast cancer, contributing toward its aggressive behaviours.

## Graphical abstract

Our space‐gene‐function study revealed four spatially distinct populations within ER^+^ breast cancers. These populations showed functional diversity (oestrogen‐responsive, proliferative, hypoxia‐induced, and inflammation‐related). The ‘proliferative’ population, rather than the ‘oestrogen‐responsive’ population, was crucial for oestrogen‐dependent tumour growth, leading to the acquisition of luminal B‐like features.

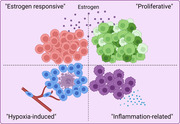

## 1 BACKGROUND

Human breast cancers were initially classified into four intrinsic subtypes: oestrogen receptor‐positive (ER^+^)/luminal, basal‐like, HER2^+^, and normal breast‐like.^1^ Later studies of the molecular portrait of human breast cancers further divided the luminal epithelial/ER^+^ tumours into two subgroups (i.e., luminal A and B) based on gene expression profiles, which showed differential clinical outcomes.^2^, ^3^ The luminal A cancers exhibit high ER‐related gene expression and low proliferation, while luminal B cancers show less expression of ER‐related genes and higher proliferation. Patients with luminal B cancers have worse prognoses. Thus, in clinical practice, a surrogate classification of luminal breast cancer is widely performed based on immunohistochemical evaluation for ER, progesterone receptor (PR; an oestrogen‐response marker) and Ki67 (a proliferative marker), serving as important criteria for therapy decisions.^4^

ER^+^ breast cancers usually depend on oestrogen for their development and progression.^5^ Although the clinical outcomes of ER^+^ breast cancers have greatly improved with the emergence of endocrine therapies, some patients, particularly those with metastatic cancers, do not respond well to such treatments.^6^ Furthermore, despite studies reporting wide ranges of ER positivity in patients, the molecular basis of endocrine responses in ‘ER‐low’ (<10% ER positivity) compared to ‘ER‐high’ tumours has not been clearly defined.^7^, ^8^, ^9^

Intratumour heterogeneity, which refers to variations of cell populations with distinct genetic, epigenetic, transcriptomic, or phenotypic profiles within a tumour lesion, is one of the hallmarks of cancers including breast cancers.^10^, ^11^ While current treatment approaches mostly target cancer as a homogeneous disease (e.g., ER antagonists used to treat ER^+^ breast cancers), intratumour heterogeneity often contributes to the acquisition of therapy resistance through the expansion of pre‐existing resistant cells.^12^ The influence of oestrogen on individual cell populations within ER^+^ breast cancers, as well as the importance of their oestrogen‐dependent growth responses have yet to be better defined.

In this study, we performed spatial transcriptomics (ST) and single‐cell RNA sequencing (scRNA‐seq) analyses on patient‐derived xenograft (PDX) models with distinct biological responses to oestrogen. Our ST analyses identified heterogeneous cell populations that were physically separated and were found in both tumours with and without oestrogen treatment. We also estimated the spatial, genetic and functional relationships of these spatially resolved cell populations within ER^+^ tumours. These cell populations were further defined by scRNA‐seq on the same experimental models. Investigations using additional scRNA‐seq datasets and dual immunohistochemistry (IHC) analysis on tumours from in vivo experiments with a CDK4/6 inhibitor supported our findings from ST analyses and characterised distinct roles and responses to oestrogen from proliferative cells in ER^+^ breast cancers. Finally, we analysed public clinical datasets using the gene signatures from the spatially distributed cell populations, exploring the potential translational value of identifying distinct functional compartments in ER^+^ breast cancers, especially with respect to luminal A and B breast cancers.

## 2 METHODS

### 2.1 ST experiments on GS3 and SC31

Two ‘ER‐high’ PDXs named GS3 and SC31, which were previously established from metastatic lesions and possessed definitive and contrasting growth responses to oestrogen, were used (Table S1). GS3 (80−100% ER) was shown to be suppressed by oestrogen treatment (‘oestrogen‐suppressed’) and to present molecular features of a luminal B cancer.^13^, ^14^ SC31 (40−90% ER) showed ‘oestrogen‐dependent’ growth and was previously defined as a luminal A cancer model despite its HER2 expression.^13^, ^14^, ^15^ Tumour pieces of GS3 and SC31 were implanted into mammary fat pads of 8−10‐week‐old NOD‐SCID/IL2Rγ^−/−^ (NSG) mice.^13^, ^14^, ^15^ Once the implanted tumours were established, placebo or 17β‐oestradiol (E2; 1 mg) pellets were implanted subcutaneously. To ensure that enough tumour mass remained for the analysis, ‘oestrogen‐suppressed’ GS3 tumours were implanted in intact mice and treated with E2 in vivo only for 7 days, whereas ‘oestrogen‐dependent’ SC31 tumours were implanted in ovariectomised mice and treated for 6 weeks, as described in our previous scRNA‐seq study.^14^ After the treatment periods, the mice were euthanised, and tumour samples were collected.

Tumour tissues were trimmed to fit the fiducial frame of Visium Spatial Gene Expression Slide (10× Genomics, PN‐2000233) while avoiding tumour core regions with large necrotic tissues. The tissue pieces were embedded on optimal cutting temperature compounds and stored at −80°C. Cryosections were prepared at 10 μm thickness, stained with haematoxylin and eosin (H&E) and captured with Zeiss Observer II (Carl Zeiss). Tissue permeabilisation was performed for 6 min as optimised using Visium Spatial Tissue Optimization Kit (10× Genomics, PN‐3000394). The sequencing libraries from each section were prepared with the Visium Spatial Gene Expression Reagent kit (10× Genomics, PN‐1000187) according to the manufacturer's instruction and sequenced with NovaSeq 6000 (Illumina). Raw sequencing data were processed using the 10× Genomics Space Ranger pipeline and Loupe browser and aligned to GRCh38 human or mm10 mouse genome.

### 2.2 ST data analyses

ST data were processed using the Seurat R package unless otherwise noted.^16^ Details for initial assessment of the ST datasets are described in Supplementary Methods. The log‐normalised data were obtained from the raw count data using the NormalizeData function and used for the cell cycle analysis (using the CellCycleScoring function) as well as visualisation of gene expressions.

The spots in the ST dataset were classified based on *ESR1*, *PGR* and *MKI67* expression (EPK classification). We evaluated bimodal expression patterns of each gene as reported in a previous study (Figure S1),^17^ and then picked a cutoff value of 0.2 to maximise the detection of positive spots. The spots were classified as ‘Negative’ or ‘Positive’ according to the cutoff expression level. Each character of the class represents the status of *ESR1*, *PGR* and *MKI67* expression (e.g., PNP represents a *ESR1*‐positive, *PGR*‐negative, *MKI67*‐positive spot).

Raw count data on each tissue were normalised with the SCTransform (SCT) function. The normalised data from the four tissues (GS3‐Placebo, GS3‐E2, SC31‐Placebo and SC31‐E2) were integrated according to the developer's vignette. Then, principal component analysis (PCA), Uniform Manifold Approximation and Projection (UMAP) dimension reduction and cluster detection with the Louvain algorithm were performed (dims = 30, res = 0.6). To identify the genes highly expressed in each cluster, the differentially expressed genes (DEGs) were analysed using the Wilcoxon rank sum test implemented in the FindAllMarkers function on the SCT normalised data (logfc.threshold = 0.25, min.pct = 0.1). The top 10 genes for each cluster were visualised using the DoHeatmap function.

To identify the gene signatures enriched in each cluster, the gene signature scores were calculated using the VISION R package^18^ and ‘hallmark’ gene sets from Molecular Signature Database.^19^ The *Z*‐score for each gene signature among clusters was calculated and visualised using the Complexheatmap R package.^20^ To assess the effect of E2 treatment on the gene signatures, the gene signature scores were compared between the individual clusters from placebo‐ and E2‐treated tissues in each model.

### 2.3 scRNA‐seq analyses

The scRNA‐seq datasets prepared from GS3‐Placebo/E2 and SC31‐Placebo/E2 in our previous study were analysed.^14^ An additional scRNA‐seq dataset on ‘ER‐low’ GS1 model (5% ER; Figure S2), which was defined as luminal B subtype in our previous publication,^13^ was prepared as described in Supplementary Methods. The numbers of cells included in the datasets were summarised in Table S2. A human ER^+^ breast cancer dataset was obtained from the Broad Institute Single Cell portal.^21^ After data integration as described in Supplementary Methods, PCA, UMAP dimension reduction and cluster detection, as well as cell cycle analysis, DEG detection, VISION gene signature scoring and EPK classification were performed as described in the ST analyses. To compare the clusters identified in the ST dataset (ST clusters) and the clusters in the scRNA‐seq dataset (SC clusters) from GS3 and SC31, Pearson's correlation coefficients of the hallmark gene signature scores were calculated between each ST and SC cluster and visualised using the corrplot R package. The absolute value of a correlation coefficient between 0 and 0.2, 0.2 and 0.4, 0.4 and 0.6, 0.6 and 0.8 or 0.8 and 1.0 were considered as ‘no’, ‘weak’, ‘moderate’, ‘strong’ or ‘very strong’ correlation, respectively. In addition, mapping of SC clusters on the ST section was performed by calculating AUCell scores^22^ of ST spots using top 50 genes from SC clusters according to a previous literature.^21^

Using the VISION R package and mammary ‘Stem’ gene set curated in our previous article,^23^ the gene signature scores of the cells in each SC cluster were calculated. For the analyses on normal epithelium, a mouse dataset established in our previous paper,^23^ which includes distinct epithelial lineages and their progenitors [i.e., basal, mammary stem cells/basal‐progenitor (MaSC/B‐pro), luminal hormone‐sensing (L‐Hor), L‐Hor progenitor (LH‐pro), luminal alveolar (L‐Alv) and L‐Alv progenitor (LA‐pro)], was obtained. The mouse orthologs of human *ESR1*/*PGR*/*MKI67* (i.e., *Esr1*/*Pgr*/*Mki67*) were used for the EPK classification as described in the ST analyses.

### 2.4 In vivo evaluation of palbociclib treatment on SC31

After the SC31 tumours were established, E2 pellets (1 mg) were implanted into the mice. Then, the mice were randomised into either E2 group (*n* = 5) or E2 + palbociclib group (*n* = 4). Palbociclib (LC Laboratories) was dissolved in phosphate‐buffered saline and administered at 50 mg/kg daily via oral gavage. After 4 weeks of treatment, the mice were euthanised and tumour samples were collected. The tumour tissues were fixed with 10% neutral buffered formalin and were embedded in paraffin.

### 2.5 Dual IHC of SC31 for ER/Ki67 and Ki67/PR

The dual IHC (ER/Ki67 and Ki67/PR) was performed by the Pathology Solid Tumor Core at City of Hope using Ventana Discovery Ultra IHC Auto Stainer (Roche Diagnostics). Primary antibodies used for the immunostaining include human ERα rabbit monoclonal antibody (Roche Diagnostics, 790−4325), human PR rabbit monoclonal antibody (Roche Diagnostics, 790−4296) and human Ki67 rabbit monoclonal antibody (Roche Diagnostics, 790−4286). Images were captured with VENTANA iScan HT (Roche Diagnostics) and analysed using the Cell Detection and Cell Classification functions implemented in Qupath software.^24^ Staining protocol details are described in Supplementary Methods.

### 2.6 Clinical outcome analysis on public cohorts with ST signatures

Molecular Taxonomy of Breast Cancer International Consortium (METABRIC) dataset^25^ was obtained from cBioPortal^26^, ^27^ using the cBioPortalData R package.^28^ ER^+^ metastatic breast cancer cohort data (GSE124647^29^) were obtained from National Center for Biotechnology Information Gene Expression Omnibus data repository. The gene signature scores on microarray data from each patient were calculated with the GSVA R package^30^ using the top 20 genes from the clusters identified in the ST analyses (Table S3). For a gene annotated with multiple probes, a mean value was calculated to represent its expression level. In the METABRIC dataset, the signature scores were compared among the breast cancer intrinsic subtypes. Then, the patients were divided into three groups based on the calculated scores, and the patients in the top and bottom tertiles were assigned as high and low groups, respectively. The patients were further classified based on the combination of ST_0 and ST_2 scores into four groups. Overall survival of each group was visualised with the Kaplan–Meier method. Details for an additional cohort [The Cancer Genome Atlas (TCGA)^31^] are described in Supplementary Methods.

### 2.7 Statistical analysis

Statistical analyses were performed using R software. Two‐group comparison was tested by Student's *t*‐test and multiple comparison was adjusted by Bonferroni correction. Multiple group comparison was performed by one‐way ANOVA followed by post hoc Tukey‐Kramer test. The difference of overall survival among the groups was tested by log rank method. *p <* .05 was considered statistically significant.

## 3 RESULTS

### 3.1 Study design

**FIGURE 1:**
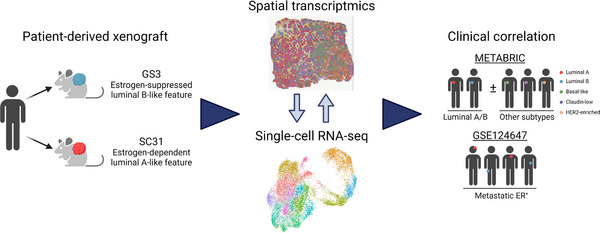
The schematic diagram of the research design in this study.

Although the clinical features of two subtypes of luminal ER^+^ breast cancers are well recognised, their molecular basis and biological origins have not yet been clearly defined. To address this gap, we designed our study using ST and scRNA‐seq on ER^+^ breast cancer PDXs (Figure 1). PDXs are recognised as valuable models for evaluating tumour responses to a given agent, in addition to underlying mechanisms of action.^32^ We previously established a set of ER^+^ breast cancer PDXs with different levels of ER positivity and varying responses to oestrogen.^13^, ^15^ Among them, we investigated two ‘ER‐high’ GS3 and SC31 models, which was suppressed by and totally depended on oestrogen treatment for their growths, respectively. Additionally, an ‘ER‐low’ GS1 model, whose growth was not totally dependent on, but accelerated by oestrogen (‘oestrogen‐accelerating’), was investigated for validating our findings from the two ER‐high tumours. These models enable us to better understand the biology of ER^+^ breast cancers as well as the mechanisms of oestrogen‐dependent tumour growth.

ST has been recognised for its potential in localising different cell populations within a tissue of interest and linking those populations to various physiological and pathological processes.^33^ With the availability of the oestrogen‐responsive ER^+^ PDXs in our laboratory, we applied ST to determine the space‐gene‐function relationship of tumour cell populations and the oestrogen response of two types of ER^+^ breast cancers, which could not be accomplished by previous studies using surgically collected clinical specimens. In addition to the lack of a direct assessment of hormone or drug response, controlling the quality and quantity of clinical specimens is often difficult, limiting the translational value of results generated from such samples.

Visium, a well‐established ST method from 10× Genomics and utilised in our study, analyses the transcriptome for each arrayed spot, which typically contains more than one cell.^34^ To compensate for the lower resolution of the ST data and to ensure reproductivity of obtained results, we also analysed scRNA‐seq data from a separate batch of PDX samples^14^ and evaluated these two datasets together. Combining both ST and scRNA‐seq analyses provides a comprehensive view of the intratumour heterogeneity while maintaining spatial arrangement of cancer cells. Although our intention was to define physically separated and functionally unique cell clusters within the two types of ER^+^ breast cancers through ST technology, we recognised that many studies utilise single‐cell datasets in tandem with ST to detect localisations of single‐cell populations in tumour tissues.^21^ Thus, for further investigation of our findings, we estimated the location of the cells from our single‐cell dataset mapped to our ST results, primarily focusing on single‐cell clusters correlating to a single ST cluster important for oestrogen‐mediated cancer growth. The results from our spatial and single‐cell analyses were further validated by similarities to a human ER^+^ breast cancer single‐cell dataset^21^ and protein‐based dual IHC of SC31 showing the importance of clinical markers co‐localisation [i.e., ER, PR and Ki67].

It is difficult to collect paired tumour samples from primary and metastatic sites especially for ER^+^ cancers, first, because the metastasis of such cancers typically take place many years later and second, there are many logistical challenges to locating primary tumours, which can be kept at separate institutions. Last, subtype changes can sometimes occur during metastasis,^35^ further complicating paired sample collection. Recognising these challenges, we have decided to evaluate the value of gene expression signatures identified in our ST dataset by analysing several clinical cohorts including luminal as well as other subtypes^25^ together with metastatic ER^+^ breast cancers.^29^ The gene signatures from our ST clusters have been shown to have prognostic values beyond ER^+^ breast cancers. As described in detail below, for the first time, cells expressing genes regulated by oestrogen were found to be physically separated from cells involved in oestrogen‐mediated proliferation. The ‘proliferative’ cell compartment was suggested to be a causative feature of luminal B cancers and shared characteristic with luminal epithelial cell progenitors, based on the analysis using curated gene signatures from a normal mammary epithelial cell atlas.^23^

### 3.2 Oestrogen dependency of ER^+^ breast cancers

To understand how different ER^+^ breast cancers respond to oestrogen, our laboratory prepared and characterised a set of ER^+^ PDXs (Table S1). These PDXs had varying levels of ER positivity and responses to E2 including: E2‐dependent (SC31,^15^ GS4), ‐accelerating (SC1,^15^ GS1, GS2) and ‐suppressed (GS3^14^) tumours (Figure S3A). These results support the clinical observation that not all ER^+^ patients respond to oestrogen and endocrine therapies in the same fashion,^6^ and that there is no direct correlation between ER levels and oestrogen responsiveness. This furthers the importance of understanding the molecular and cellular mechanisms of oestrogen activity in tumours/tumour models with different response patterns, which can lead to more effective targeted therapies.

‘Oestrogen‐suppressed’ cancers are uncommon, but they have been found clinically and linked to aromatase inhibitor resistance, like our GS3 which was established from a patient who failed such treatment. A recent clinical trial has been launched aimed at investigating the oestrogen‐suppressed features of endocrine‐resistant cancer.^36^ Our study included the oestrogen‐suppressed GS3, which can be a valuable tool to characterise oestrogen‐mediated growth by comparing it to the oestrogen‐dependent SC31.

### 3.3 ST on two ER^+^ breast cancer PDX models with opposite responses to oestrogen

To identify phenotypical diversity within ER^+^ breast cancers and to reveal how E2 affects their growths, we performed ST analyses on ‘ER‐high’ GS3 and SC31, both treated with and without E2 (Figure 1). Although PDX models are known to have ‘phenotypic drift’,^37^ growth suppression of GS3 and promotion of SC31 by E2 were maintained over the passages used in this study (Figure S3B).

To assess the quality of our ST experiments, we first evaluated the H&E images of tissue sections (Figure S4). GS3 sections mostly consisted of tumour cells and small patches of stromal and/or necrotic areas. In SC31 sections, we observed both tumour cells and scar‐like areas which were possibly derived from the replacement of necrotic tissues during tumour growth. In the datasets obtained from the four tissue sections, a total of 9323 spots were included (2330 spots from GS3‐Placebo, 2863 spots from GS3‐E2, 1967 spots from SC31‐Placebo and 2163 spots from SC31‐E2) with an average number of 19 327 nCount and 4824 nFeature for human genes. Although the spots located on the necrotic and/or scar‐like areas showed lower values of both nCount and nFeature, the spots consisting of tumour cells had better quality control (QC) metrics (i.e., higher nCount/nFeature and low mitochondrial gene %; Figure S5), confirming that the overall experimental procedures were successful.

A well‐recognised challenge of PDXs is the presence of mouse stromal cells, preventing investigation into the original tumour microenvironment.^37^ In the ST datasets, we observed mouse‐derived stromal cell marker expressions (Figure S6), but almost no human‐derived stromal cell marker expressions (Figure S7). However, less than 5% of the spots included more mouse than human genes (Figure S8). Furthermore, ‘mouse’ spots (spots with more mouse than human genes) were mostly located near the edge of tumour areas. These results indicated that most of the cells making up the ST spots in our dataset were human‐derived epithelial cells. Our previous and current studies showed that the effect of oestrogen on the chosen PDXs has been maintained in multiple passages,^14^, ^15^ suggesting that the oestrogen‐dependent growth in our model depends on the epithelial cells of the originated tumour tissues and that the impact of mouse stromal cells was minimal. Therefore, in this study, we focused our analyses on the human transcriptome from the epithelial cells.

**FIGURE 2:**
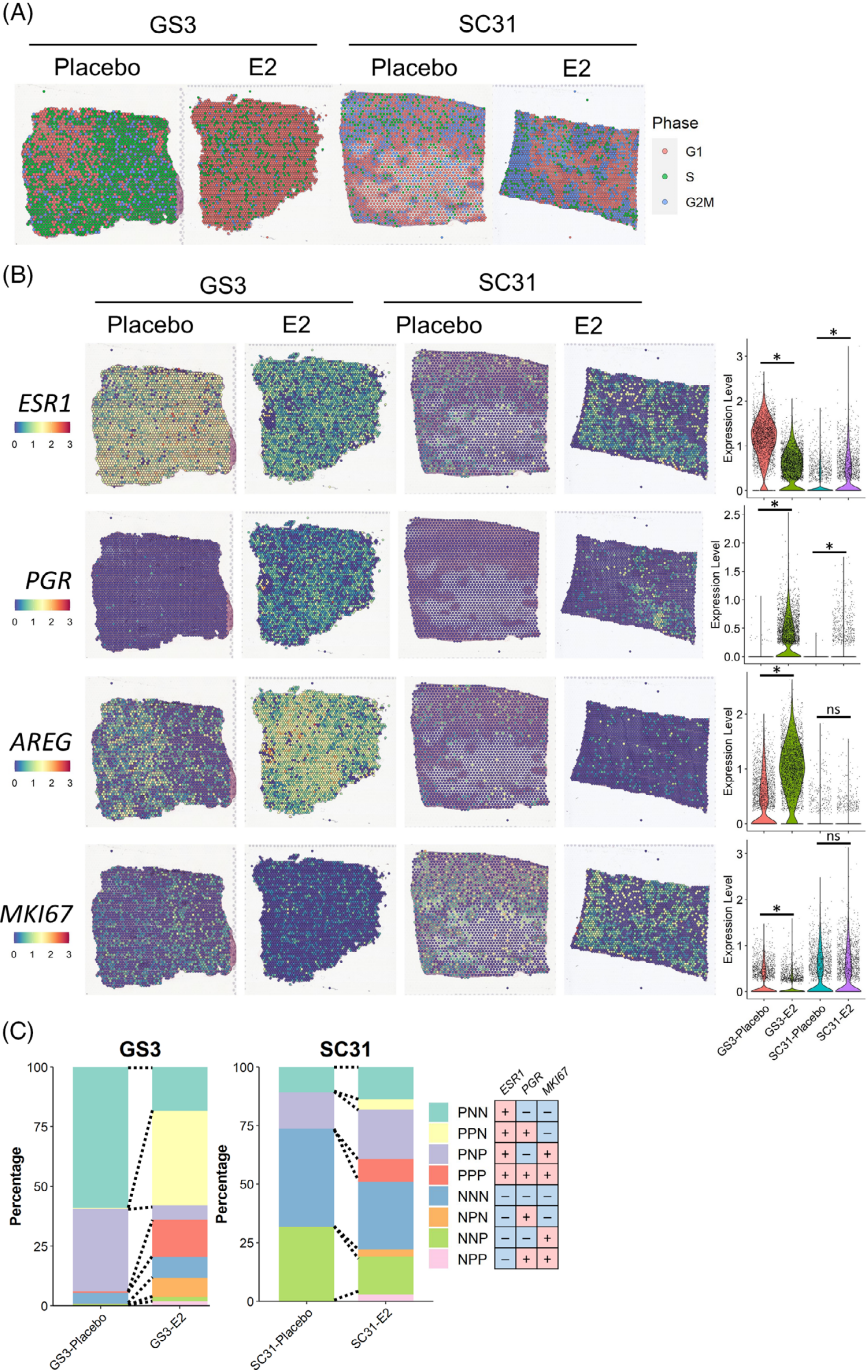
Spatial transcriptomics on two ER^+^ breast cancer PDX models. (A) Cell cycle analysis on the ST datasets from GS3 and SC31 with and without E2 treatment. The colour of each spot represents the cell cycle phase. (B) Expression of *ESR1*, *PGR*, *AREG* and *MKI67* genes in the ST datasets. The spatial plot on the left shows the location and level of the expression on each tissue. The violin plot on the right summarises the gene expressions in each section. *, *p* < .05; ns, not significant. (C) EPK classification on the ST datasets.

To confirm the oestrogen‐mediated tumour responses observed in our previous studies^14^, ^15^ as well as to get a brief insight of spatial gene expression pattern in our ST datasets, we performed cell cycle analysis and examined the expressions of *ESR1, PGR*, and *MKI67* genes (encoding the three important markers ER, PR, and Ki67, respectively) (Figures 2A and B). Our results indicated that the proportion of proliferating spots (in S and G2M phases) greatly decreased in GS3 and increased in SC31 with E2 treatment, confirming that our ST datasets reflected the discrete tumour growth responses of these PDX models (Figures 2A and S9). Furthermore, *MKI67* expression decreased in GS3 following E2 treatment (Figure 2B). We found that the number of spots expressing *ESR1* and their expression levels decreased in GS3 and increased in SC31 in E2‐treated specimens, consistent with our previous results.^14^ Remarkably, *PGR* expression was found in both GS3 and SC31, but only when treated with E2. For comparison, another well‐known oestrogen‐regulated gene, *AREG*, was found in tumours without E2 treatment, especially in GS3, and was up‐regulated by E2 treatment. Although known oestrogen‐regulated genes can be expressed in the absence of oestrogen, the expression of *PGR* was suggested to be entirely oestrogen‐dependent. These observations are important to indicate that oestrogen‐regulated genes, such as *PGR*, can be up‐regulated independently from the differential oestrogen‐mediated tumour growth responses. *IL24*, encoding for an oestrogen‐induced suppressor as indicated in our previous report,^14^ was only detected in GS3‐E2, supporting the conserved features of our PDX models (Figure S10). Intriguingly, the expression levels of these gene markers were not consistent throughout spots on the tissues, which reflected intratumour heterogeneity even among the canonical marker expressions of luminal breast cancers. Considering that the changes in *ESR1*/*PGR*/*MKI67* expression in the ST dataset were comparable to IHC analysis in our previous study,^14^ we then classified each spot by their *ESR1*/*PGR*/*MKI67* gene expression patterns (Figure 2C). Again, in both GS3 and SC31, the proportions of *PGR* ^+^ spots, such as *ESR1* ^+^ *PGR* ^+^ *MKI67* ^−^ (PPN; each letter representing the positive or negative expression of each gene) spots, were increased by E2 treatment. Strikingly, only the changes in the proportion of PNP spots (i.e., a decrease in GS3 and an increase in SC31) were correlated with the suppression of GS3 and promotion of SC31 by E2 treatment, respectively. Overall, these results demonstrated that the well‐established markers (i.e., *ESR1*, *PGR*, and *MKI67*) showed distinct spatial distributions and that the cells included in PNP spots (i.e., *ESR1* ^+^ and/or *MKI67* ^+^ cells) were potentially associated with oestrogen‐mediated tumour growth.

### 3.4 ST identified functional heterogeneity in ER^+^ breast cancers

**FIGURE 3:**
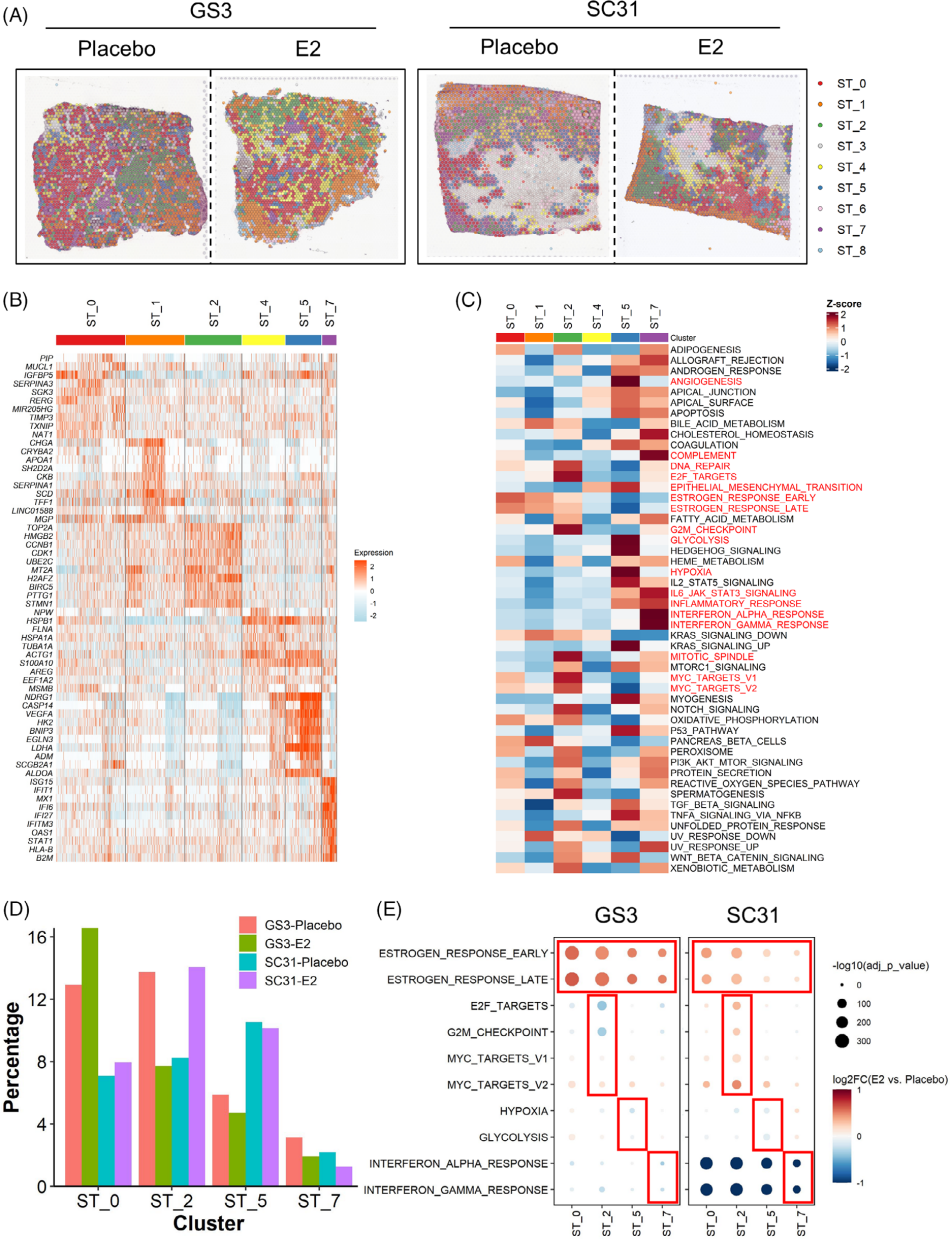
Identification of functional compartments in the ST dataset and the effect of E2 treatment. (A) Distribution of the ST clusters on tumour sections. Each spot is coloured according to the cluster (ST_0−8) identified by the unbiased clustering. (B) Heatmap of the top 10 genes for the ST clusters. Column represents the spots and row represents the genes. The gene expression levels were scaled by the SCTransform function. (C) Heatmap of the hallmark gene signature scores for the ST clusters. Column represents the ST clusters and row represents the gene sets. (D) Proportion of the four major ST clusters in each sample. The percentage of the ST cluster was calculated by (the number of spots in a ST cluster in a sample)/(the total number of spots in a sample). (E) Gene signature changes by E2 treatment. Size and colour of the dot represent *p* value and log2 fold change value between E2 and placebo groups in each ST cluster, respectively.

To further explore the intratumour heterogeneity in ER^+^ breast cancers and to reveal cell population(s) that drive oestrogen‐mediated growth, we performed unsupervised clustering on the integrated dataset from the four ST samples. We identified nine clusters (ST_0−8) with diverse gene expression profiles (Figures 3A and S11A). Among the identified nine clusters, ST_3, ST_6, and ST_8 showed lower QC metric values (Figure S11B) and were mainly located in the necrotic and/or scar‐like tissue areas (Figures 3A and S12). Thus, they were not interrogated in further analyses. The other clusters (ST_0, ST_1, ST_2, ST_4, ST_5 and ST_7) were localised in the tumour areas, indicating that these clusters represented the heterogeneous populations of human breast cancer cells (Figures 3A and S12). Each cluster was identified across the four samples and tended to accumulate in a different compartment on the tumour sections (e.g., ST_0 spots on GS3‐Placebo at the left area, whereas ST_2 at the right area). In addition, ST_5 seemed to be adjacent to the necrotic areas across the four samples. Importantly, our results showed intratumour heterogeneity of breast cancer cells which was shared irrespective of the different growth responses and presence of E2 treatment.

To characterise the populations identified in our integrated dataset, we investigated the DEGs and gene signatures in each ST cluster (Figures 3B and C). ST_0 showed higher expressions of known oestrogen‐regulated genes such as *SERPINA3*, *SGK3* and *RERG*.^38^, ^39^, ^40^ Intriguingly, *TXNIP* and *RERG* have also been reported to be linked to breast cancer suppression associated with better prognosis.^40^, ^41^, ^42^, ^43^ Among the six main clusters, ST_0 also presented higher scores for the oestrogen‐response gene sets (ESTROGEN_RESPONSE_EARLY/LATE), suggesting that ST_0 is the oestrogen‐responsive population (Figure S13). The DEGs of ST_2 included many proliferation‐related genes (e.g., *TOP2A*, *HMGB2* and *CDK1*). ST_2 had the highest scores in the gene signatures related to cell cycle progression (DNA_REPAIR, E2F_TARGET, G2M_CHECKPOINT and MITOTIC_SPINDLE) as well as the highest percentage of the proliferating spots in the cell cycle analysis (Figure S14). They also showed the highest scores in MYC target signatures (MYC_TARGETS_V1/V2), which are known to be involved in breast cancer proliferation.^44^ Additionally, ST_2 showed higher scores for oestrogen‐response gene sets among the six clusters, implying that they were important in oestrogen‐dependent tumour proliferation.

ST_5 was characterised by higher expression of genes related to hypoxia and the resulting anaerobic cell metabolism or angiogenesis (e.g., *NDRG1, LDHA* and *VEGFA*), as well as their associated gene signatures (ANGIOGENESIS, EPITHELIAL_MESENCHYMAL_TRANSITION, GLYCOLYSIS and HYPOXIA). In previous studies, these features were reported to be related to breast cancer metastasis.^45^, ^46^ Considering the localisation of this cluster along with the necrotic/scar‐like areas, ST_5 would represent the subset of cancer cells exposed to hypoxic microenvironments and thereby obtaining metastatic features. ST_7 showed higher gene expression of interferon (IFN)‐inducible genes such as *ISG15*, *IFIT1* and *IFI6*, which were recently demonstrated to be associated with ER^+^ breast cancer therapy resistance.^47^, ^48^, ^49^ The ST_7 cluster also showed the highest scores in the signatures related to inflammation (e.g., COMPLEMENT, IL6_JAK_STAT3_SIGNALING, INFLAMMATORY_RESPONSE and INTERFERON_ALPHA and GAMMA_RESPONSE). Although ST_1 showed higher expression of some oestrogen‐inducible genes (e.g., *TFF1*) and the oestrogen‐response gene signatures, their overall gene signature scores were lower than other clusters. The relatively lower scores were also observed in ST_4, implying that ST_1 and ST_4 would be functionally less distinct compared with the others. Collectively, the ST analyses revealed the four major spatially resolved ‘functional compartments’ with important gene signatures identified in all four tumour sections.

### 3.5 Impact of oestrogen on the individual functional compartments

Our ST analyses identified the heterogeneous populations in either breast cancer models, regardless of oestrogen treatment. To assess the impact of E2 treatment on the individual populations, we compared the proportion of the four compartments (ST_0, ST_2, ST_5 and ST_7) between placebo‐ and E2‐treated samples in GS3 and SC31 (Figure 3D). ST_2 differed in accordance with the growth of the PDX models (i.e., decreased in GS3 and increased in SC31). In contrast, the proportions of ST_0, which was the compartment with the high oestrogen‐response signature, was increased by E2 treatment in both models. Meanwhile, the abundance of ST_5 and ST_7 spots showed less changes by E2.

We then evaluated the effect of oestrogen on gene expression profiles within each of the four major ST compartments by comparing the gene signature scores between the placebo‐ and E2‐treated samples (Figure 3E). In ST_2, the levels of gene expression associated with E2F_TARGETS and G2M_CHECKPOINT decreased in GS3 and increased in SC31 following E2 treatment. The levels of genes associated with ESTROGEN_RESPONSE_EARLY/LATE increased by E2 in both tumours. MYC_TARGETS_V1/V2 scores were highly increased in SC31 compared with that in GS3, suggesting the importance of these responses for inducing oestrogen‐dependent tumour proliferation. In ST_0, the levels of genes associated with ESTROGEN_RESPONSE_EARLY/LATE were increased by E2 in both models, while the proliferative features were not changed, suggesting that the cells in ST_0 would not be linked to E2‐dependent tumour growth. The levels of genes associated with HYPOXIA and GLYCOLYSIS in ST_5 of both tumours were not prominently affected by E2, but ESTROGEN_RESPONSE_EARLY/LATE scores increased. While the major gene signatures in ST_7 were INTERFERON_ALPHA and GAMMA_RESPONSE, E2 treatment decreased these signatures in the two models, especially in SC31. In summary, the scores of the oestrogen response signatures increased across the ST clusters with E2, indicating that E2 treatment induced the typical oestrogen‐regulated gene expressions in most tumour cells in ER^+^ breast cancers, where both *ESR1^+^* and *ESR1^−^* cells would be affected as reported in our previous literature.^14^ However, only the cells in ST_2 were associated with tumour growth.

### 3.6 Single‐cell RNA sequencing defined the heterogeneous breast cancer population at a higher resolution

**FIGURE 4:**
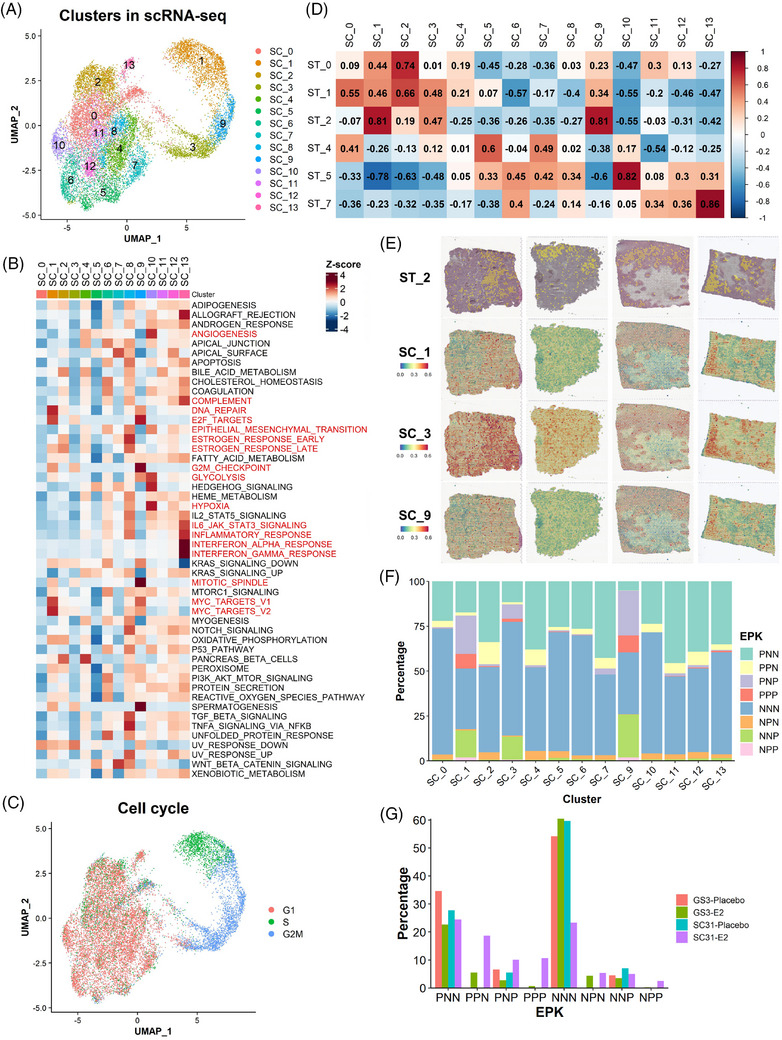
Single‐cell analysis defined cell subsets responsible for oestrogen‐dependent tumour growth at a higher resolution. (A) UMAP plot of the integrated scRNA‐seq dataset coloured according to the clusters identified by the unbiased clustering. (B) Heatmap of the hallmark gene signature scores for the SC clusters. Column represents the SC clusters and row represents the gene sets. (C) Cell cycle analysis on the integrated scRNA‐seq dataset. (D) Correlation analysis of the gene signature scores between the clusters in the ST and scRNA‐seq datasets. The value and colour of each box indicates Pearson's correlation coefficient. (E) Spatial mapping of the proliferative SC clusters on the ST sections. The spatial plot on the top represents the localisation of ST_2 spots in each tissue. (F) EPK classification on the scRNA‐seq dataset. (G) Proportion of the cells with EPK classification in each scRNA‐seq sample. The percentage of cells was calculated by (the number of cells in an EPK classification in a sample)/(the total number of cells in a sample).

Upon the identification of the heterogeneous populations in our ST dataset, we examined the scRNA‐seq datasets from the same experimental settings^14^ to determine whether each ST cluster can be recognised as an individual single‐cell population or as a mixture of several populations. After the integration of the four datasets, 14 single‐cell clusters (SC_0‐13) were identified (Figures 4A and S15A). Despite the regression for stress response‐related ‘immediate‐early gene (IEG)’ expressions (Table S4 and Supplementary Methods), a small cluster with the high expression of IEGs (SC_8) persisted, and therefore, we did not interrogate this cluster to avoid potential artifacts in further downstream analyses (Figure S15B). Remarkably, the results of DEG analyses indicated that several SC clusters had comparable gene expression patterns with the ST clusters (Figures S15B and C); the top DEGs in ST_2 (*HMGB2*), ST_5 (*NDRG1*) and ST_7 (*ISG15*) were also highly expressed in SC_1/3/9, SC_10 and SC_13, respectively (Figure S15C). On the UMAP plot, there were three well‐separated clusters, SC_1, SC_3 and SC_9, which showed highly proliferative signatures (Figures 4A and B and S15B). Corresponding to the difference of their proliferative signature patterns (e.g., high DNA_REPAIR in SC_1 and high G2M_CHECKPOINT in SC_9, respectively), these clusters were further distinguished by their cell cycle phases: SC_1 in S phase, while SC_3 and SC_9 in G2M phase (Figures 4B and C). As reported in a previous study,^50^ the levels of *MKI67* expression tended to increase along with the cell cycle progression and were the highest in G2M phase cells (i.e., SC_9; Figure S16). SC_1 had the highest levels of oestrogen response signatures among the three clusters, implying that each cluster had different contributions toward oestrogen‐dependent growth.

To compare the clusters identified in the ST and scRNA‐seq datasets, we analysed the correlation of the gene signature scores between the ST and SC clusters (Figure 4D). Corresponding to the similarity of the DEG and gene signature results, strong to very strong positive correlations were observed between ST_0 and SC_2 (oestrogen‐responsive), ST_2 and SC_1/9 (proliferative), ST_5 and SC_10 (hypoxia‐induced), as well as ST_7 and SC_13 (inflammation‐related). These correlations highlighted the most dominant single‐cell populations within the ST compartments. In addition, the ST clusters correlated with more than one SC cluster, indicating that the SC clusters defined cells included in each ST spot clusters at a higher resolution. ST_2 had very strong correlations with both SC_1 and SC_9 as well as a moderate correlation with SC_3, all of which depicted highly proliferative features. To confirm this observation, we performed the mapping of the SC clusters on the spatial datasets following the approach by Wu et al.^21^ In general, the areas showing the enrichment of SC_1/3/9 corresponded to the localisation of ST_2 spots, with minor variability (Figure 4E). Also, the predicted abundance represented by the AUCell scores of SC_1/3/9 signatures were significantly higher in ST_2 cluster (Figure S17). These results suggested that the ST_2 compartment would consist of three different proliferative cell subtypes. Altogether, we defined transcriptionally different compartments of breast cancer cells from the ST analysis and validated the concept that ST populations can be identified as distinct groups of cells, or their mixtures, using scRNA‐seq datasets at a higher resolution.

### 3.7 Further definition of cell subsets responsible for oestrogen‐dependent tumour growth with *ESR1*/*PGR*/*MKI67* expressions

As observed in the ST dataset (Figure 2B), the global expression pattern of these three genes in each dataset correlated with our previous IHC observation (Figure S18).^14^ Therefore, to link the expression pattern of the canonical luminal breast cancer markers with the ‘proliferative’ ST_2‐associated SC clusters, we further investigated our scRNA‐seq datasets using the classification based on *ESR1*/*PGR*/*MKI67* expressions (Figure 4F). In the evaluation of the proportion of each classification in the SC clusters, the *MKI67^+^* cells (PNP, PPP, NPP and NNP) were specifically distributed among the proliferative SC clusters (SC_1, SC_3 and SC_9). The results indicated that the *MKI67^+^* cells represented the proliferative populations contributing to the oestrogen‐dependent tumour growth. In addition, SC_1 and SC_9 had more *ESR1^+^*/*MKI67^+^* cells (PNP and PPP), while SC_3 tended to have fewer of those cells, highlighting their differences in the oestrogen responsive signatures. Meanwhile, *PGR* ^+^ and *MKI67^−^* cells, including PPN and NPN, were present in all clusters except SC_1, SC_3 and SC_9, implying that most *PGR* ^+^ cells were not associated with proliferation.

To evaluate the effect of E2 on each group of EPK‐classified cells, we analysed the changes in their proportions by E2 treatment in our scRNA‐seq dataset (Figure 4G). PPN cells were only identified in E2‐treated samples, ensuring that *PGR* expression was solely dependent on the presence of oestrogen. Meanwhile, the changes in PNP cells correlated with the response of GS3 and SC31 to E2 treatment. Although PPP/NPP cells were observed in both models with E2 treatment, the increase was much more prominent in SC31 than that in GS3, also suggesting their roles in the proliferative response to oestrogen. Accordingly, our results implicated that PNP, as well as PPP/NPP cells, are subsets of *MKI67^+^* proliferative cells crucial for oestrogen‐dependent breast cancer growth, which correlated with the results from our ST analyses.

### 3.8 Validation of the results from SC31 and GS3 through single‐cell analysis on another PDX (GS1) and publicly available human breast cancer datasets

**FIGURE 5:**
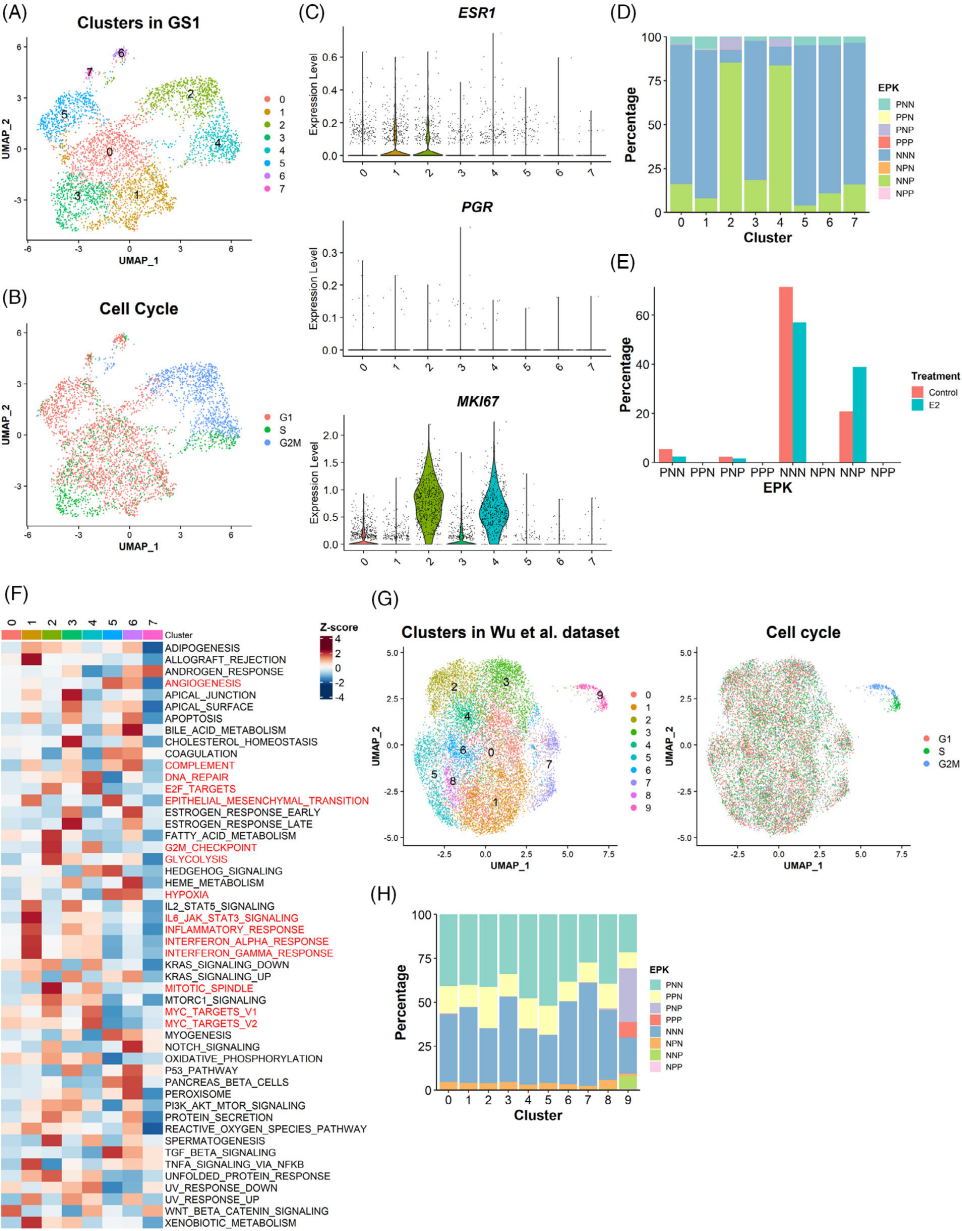
Validation of the results from SC31 and GS3 through single‐cell analysis on two additional datasets. (A) UMAP plot of the GS1 dataset coloured according to the clusters identified by the unbiased clustering and (B) the cell cycle phases. (C) Expression of *ESR1*, *PGR* and *MKI67* expression in each cluster identified in the GS1 dataset. (D) EPK classification on the GS1 dataset. (E) Proportion of cells with EPK classification in each sample. The percentage of cells was calculated by (the number of the cells in an EPK classification in a sample)/(the total number of the cells in a sample). (F) Heatmap of the hallmark gene signature scores for the clusters in GS1. Column represents the clusters and row represents the gene sets. (G) UMAP plot of human ER^+^ breast cancer dataset coloured according to the clusters identified by the unbiased clustering and the cell cycle phases. (H) EPK classification on the human ER^+^ breast cancer dataset.

To validate what we have learned from the analyses on GS3 and SC31 PDXs, we performed scRNA‐seq analysis on another PDX model named GS1. This ‘ER‐low’ (5% ER) model was defined as a luminal B cancer in our previous literature^13^ and its growth was partly facilitated by E2 (but not requiring E2), defining GS1 as an oestrogen‐accelerating luminal breast cancer model (Table S1 and Figure S3). In the scRNA‐seq dataset from GS1, the *MKI67* ^+^ cells accumulated in the two clusters with S and/or G2M phase cells (clusters 2 and 4) (Figures 5A–C), similar to those of SC_1, SC_3 and SC_9 (in the GS3 and SC31 dataset) (Figures 4C and S16). There were a limited number of cells expressing either *ESR1* or *PGR* in GS1. When comparing the EPK classification of this dataset (Figure 5D), NNP cells were more dominant than PNP cells in the proliferative clusters and the number of NNP cells increased after E2 treatment (Figure 5E), indicating that tumour growth in GS1 would be driven mainly in an ER‐independent manner, but accelerated in the presence of E2. Importantly, we identified the clusters with gene signatures related to hypoxia (cluster 5) and inflammatory responses (cluster 1), confirming the ST_5 and ST_7‐like cells in this breast cancer (Figure 5F).

To support the availability of a function‐specific ‘proliferative’ cell population for the oestrogen‐dependent growth, we investigated an additional scRNA‐seq dataset from nine ER*^+^* breast cancer patient samples.^21^ Among clusters identified in this dataset, cluster 9 showed proliferative characteristics (Figure 5G), although oestrogen response information was not available for these samples, and functional features of the other clusters were unclear due to varying sample quality and abundance of cancer cells (Figures S19A–C). EPK classification showed that the *MKI67^+^* cells specifically accumulated in the proliferative cluster 9 (Figure 5H), which supported the results from our own datasets (Figure 4F). Importantly, *PGR^+^* (e.g., PPN) cells were identified across all clusters regardless of different proliferative features, ensuring that *PGR* expression was not an indicator of proliferative capacity of the cells.

### 3.9 Analysis of ER, PR and Ki67 expression in SC31 through dual IHC following E2 and/or palbociclib treatment

While three United States Food and Drug Administration‐approved CDK4/6 inhibitors are mainly used for ER^+^ breast cancer in combination with endocrine therapy, a recent study has demonstrated single‐agent activity of a newer CDK4/6 inhibitor, abemaciclib, in the treatment of ER^+^ metastatic breast cancer.^51^ In a previous publication on ER^+^ cell lines,^52^ we have found that cell cycle‐driven ER is required for CDK4/6 inhibitor‐mediated suppression of cell proliferation, and changes in G2M‐phase molecules are associated with the treatment of CDK4/6 inhibitors. Ki67 is known to have the highest expression in cells in the G2M‐phase.^50^ CDK4/6 inhibition leads to G1‐phase arrest, reducing cells in the G2M phase, that is, Ki67^+^ cells.

**FIGURE 6:**
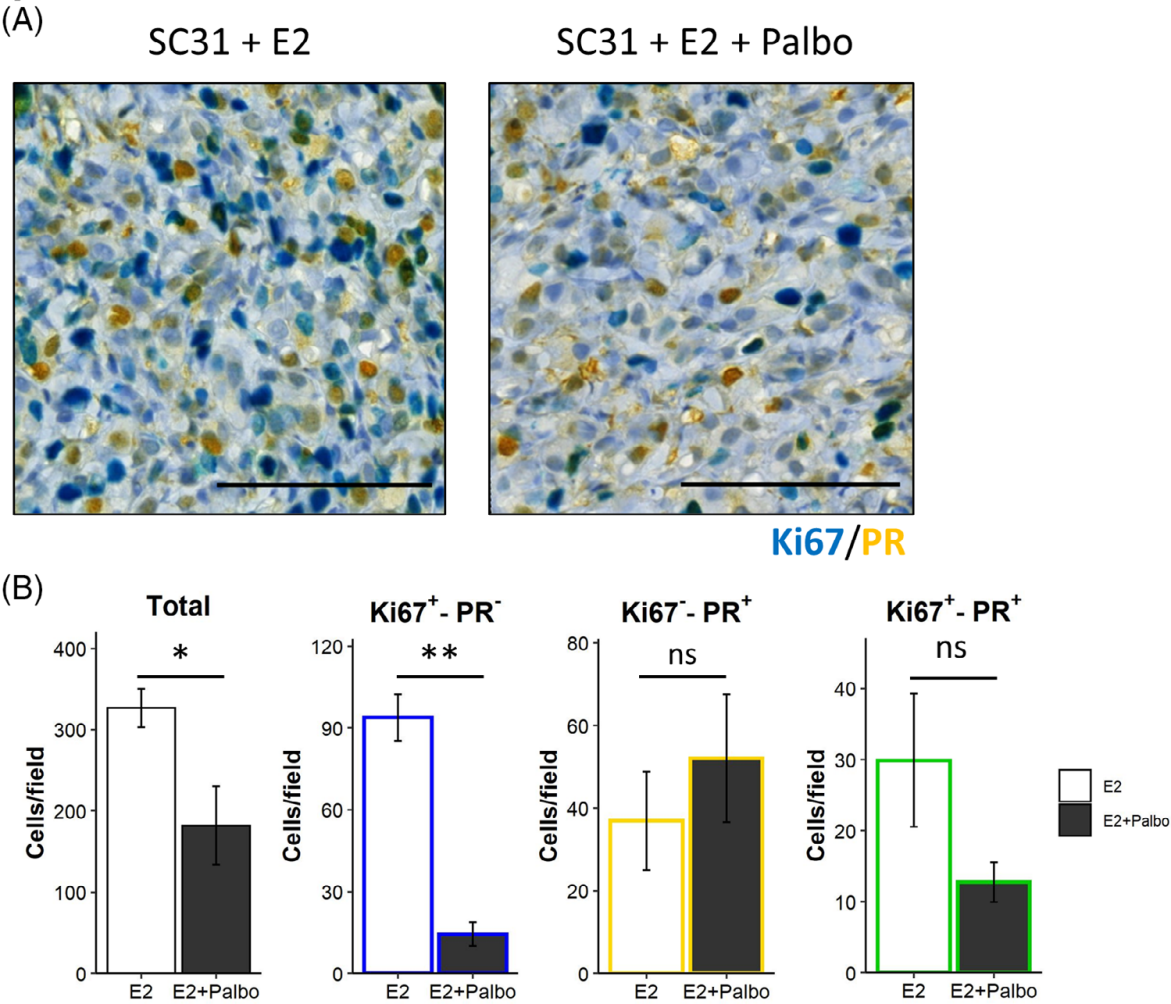
Dual IHC analysis on SC31 following E2 and/or palbociclib treatment. (A) Representative images of the dual IHC of Ki67 (blue) and PR (yellow) in SC31 treated with E2 or E2 + palbociclib (Palbo). The Ki67^+^PR^+^ cells were stained in green. Scale bar = 100 μm. (B) Quantification of the total and Ki67^+^PR^−^, Ki67^−^PR^+^ and Ki67^+^PR^+^ cells per field. Data are shown as mean ± SEM [*n* = 5 (E2) and 4 (E2 + Palbo)]. *, *p* < .05; **, *p* < .01; ns, not significant.

Here, to validate the implication of *ESR1*/*PGR*/*MKI67* expressions at the protein level and evaluate the impact of targeted proliferative cell suppression, we performed an in vivo experiment using SC31 treated with E2 and a CDK4/6 inhibitor, palbociclib (Figures 6 and S20). The histological evaluation showed that the palbociclib treatment significantly reduced the total number of the cells per area (Figure 6), indicating that palbociclib successfully reduced tumour cell growth. In our dual IHC analysis, we could confirm the identification of each EPK‐classified cell type in the ST and scRNA‐seq datasets and to link their distribution to palbociclib treatment response (Figures 6 and S20). With the palbociclib treatment, the dual ER/Ki67 IHC revealed that the number of both ER^+^Ki67^+^ and ER^−^Ki67^+^ cells were significantly reduced (Figure S20A). Furthermore, in Ki67/PR dual IHC, Ki67^+^PR^‐^ cells, which would include both NNP and PNP cells, were remarkably decreased (Figures 6B and S20B). Surprisingly, the number of Ki67^+^PR^+^ cells, including both NPP and PPP cells, was not significantly reduced by palbocilib, suggesting that palbociclib may not suppress these two classifications of cells, or that PR expression is linked to the non‐proliferative features. Overall, these observations provided protein‐based evidence for the ST/scRNA‐seq results that the induction of the population with typical oestrogen responses (e.g., Ki67^−^PR^+^ cells) was not directly linked to the oestrogen‐dependent growth and indicated that the therapeutic effect of palbociclib could be exerted by suppressing PNP and NNP cells.

### 3.10 Translational application of the four major functional compartments in ER^+^ breast cancers

**FIGURE 7:**
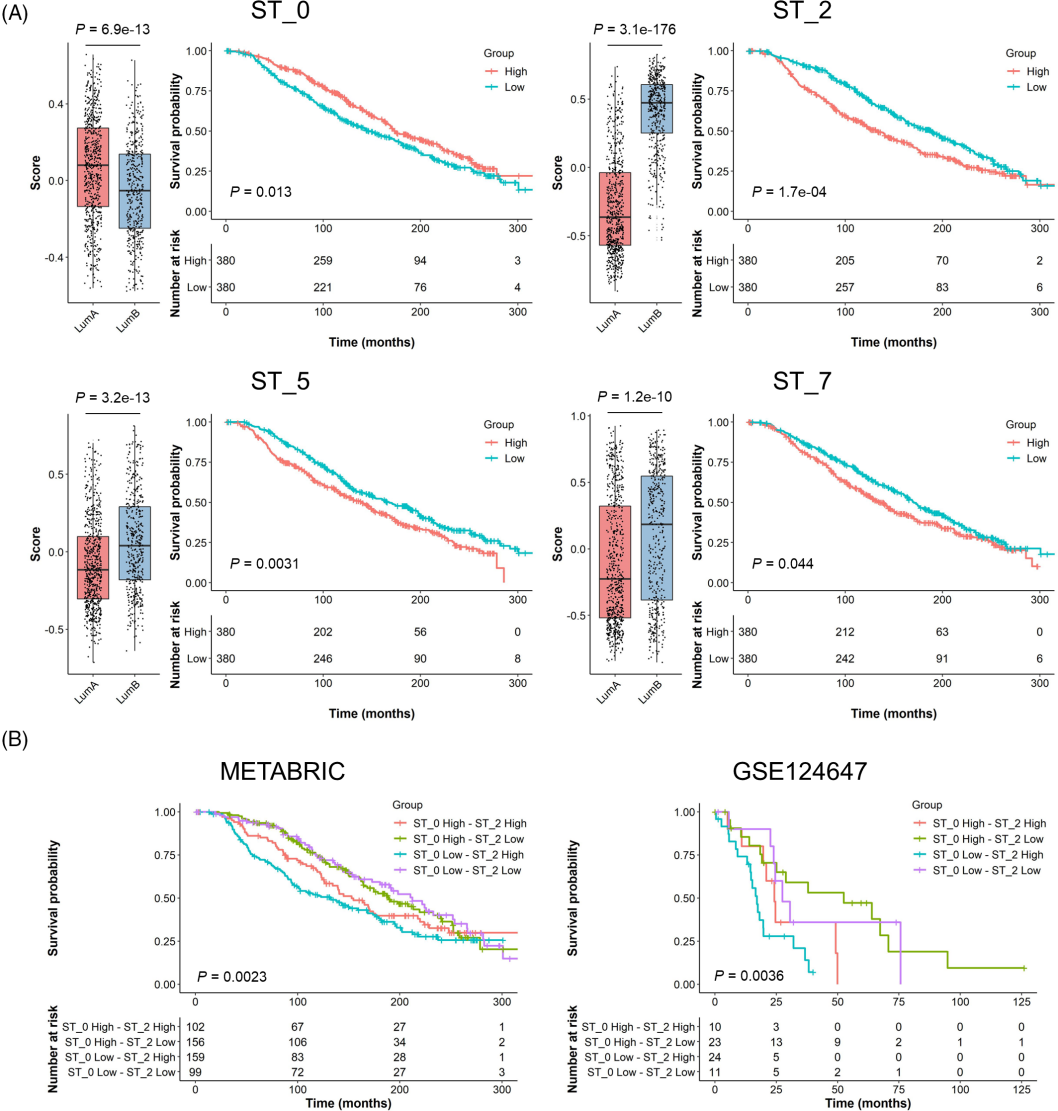
Clinical data analysis on public cohorts with the ST signatures. Luminal subtype breast cancers in METABRIC cohort (A and B; luminal A, *n* = 679; luminal B, *n* = 461) and stage IV ER^+^/HER2^−^ breast cancers in GSE124647 cohort (B; *n* = 140) were analysed using A ST_0, ST_2, ST_5 or ST_7 signatures and B the combination of ST_0 and ST_2 signatures. The gene signature scores were calculated in each patient using the GSVA R package. Left panels on A show the scores in luminal A and B patients with *p* values on the top. Kaplan–Meier plots show the overall survival of patients in each group with *p* values at the bottom left corner.

To provide clinical implications of our findings on the four ST clusters, we analysed a publicly available dataset, METABRIC, using the gene signatures from ST_0, ST_2, ST_5 and ST_7 compartments (Figure 7A). Among the luminal breast cancer patients, the ST_0 signature scores were higher in the luminal A patients, while the scores of the other signatures (from ST_2, ST_5 and ST_7) were significantly higher in the luminal B patients. The difference of the ST_2 signature scores was highly significant, which agreed with the molecular definition of luminal B subtype as a highly proliferative ER^+^ breast cancer. Patients in the high score group of ST_2, ST_5 and ST_7 signatures had significantly shorter survival, suggesting that these populations were potentially associated with more aggressive features of ER^+^ breast cancers. On the other hand, patients with the high ST_0 scores showed better prognoses, confirming that ST_0 would not be directly associated with oestrogen‐dependent tumour progression.

Considering the importance of ST_0 and ST_2 signatures (i.e., oestrogen response and proliferation) in luminal cancers and their opposite association with patient survival, we carried out an analysis combining these two gene signatures (Table 1 and Figure 7B). The tumours with low ST_2 signature scores were primarily luminal A subtype with better prognosis, while the ST_0 signature scores did not exhibit much impact. Furthermore, the tumours with high ST_2 scores, especially ST_0 low‐ST_2 high tumours, were mainly luminal B subtype with shorter survival times. We performed a similar comparison using the TCGA data set (Table 1). The ST_2 low tumours were all luminal A subtype except for one tumour. ST_0 scores barely affected the luminal subtype distribution. We also examined a third dataset (GSE124647) that included 140 metastatic ER^+^/HER2^−^ breast cancers.^29^ While this dataset did not have intrinsic subtype information, high ST_0 and ST_2 scores were associated with better and worse survival outcomes, respectively (Figure S21), the same as those observed in the METABRIC dataset (Figure 7A). Similarly, ST_2 low tumours had a better outcome and ST_0 low‐ST_2 high tumours had the shortest survival times (Figure 7B). The results from these analyses suggest that the spatially distinct ST_2 group (including *MKI67^+^* cells) had more impact than ST_0 on luminal cancer prognoses, and that luminal B tumours could be associated with a higher abundance of cells belonging to the ST_2 compartment. It was also clear that luminal A tumours had low ST_2 scores.

**TABLE 1:** ST_0 and ST_2 combination groups and luminal subtypes in the public cohorts.

	METABRIC		TCGA	
	Luminal A	Luminal B	Luminal A	Luminal B
ST_0 High—ST_2 High	27 (26%)	75 (74%)	23 (42%)	32 (58%)
ST_0 High—ST_2 Low	152 (97%)	4 (3%)	103 (99%)	1 (1%)
ST_0 Low—ST_2 High	22 (14%)	137 (86%)	21 (22%)	73 (78%)
ST_0 Low—ST_2 Low	96 (97%)	3 (3%)	64 (100%)	0 (0%)

Further investigation on the entire METABRIC cohort including other subtypes (i.e., basal‐like, claudin‐low and HER2‐enriched) showed that luminal A and B had higher scores in ST_0 signature than the other subtypes, and again, the clinical outcome of high score group was better than the low group's (Figure S22). For ST_2 signature, luminal A was the lowest, and luminal B had relatively higher scores amongst the subtypes. Although ST_2 was characterised as an oestrogen‐responsive and proliferative population, this signature was also high in the basal‐like subtype because the genes included in ST_2 signature consisted of many proliferation‐related genes. ST_5 score was significantly higher in the basal and HER2 tumours, which depicted their more aggressive and metastatic behaviours as well as oestrogen‐independent features. Also, higher ST_5 score indicated worse outcomes, supporting the potential contribution of ST_5 to cancer metastasis through oestrogen‐independent mechanisms. The differences of ST_7 signature scores among the subtypes were also significant but less clear than the other signatures. In addition, the levels of the ST_7 signature did not show significant value on the survival of all breast cancer patients. It may indicate that the activation of their inflammation‐related features, especially IFN‐responsive characters, was only related to the aggressiveness of luminal breast cancers. In summary, our analysis on the publicly available cohorts with bulk gene expression information associated the functional ST compartments with clinical outcomes, furthering the translational value of the results from our ST analyses on the oestrogen‐responsive ER^+^ breast cancer PDXs.

**FIGURE 8:**
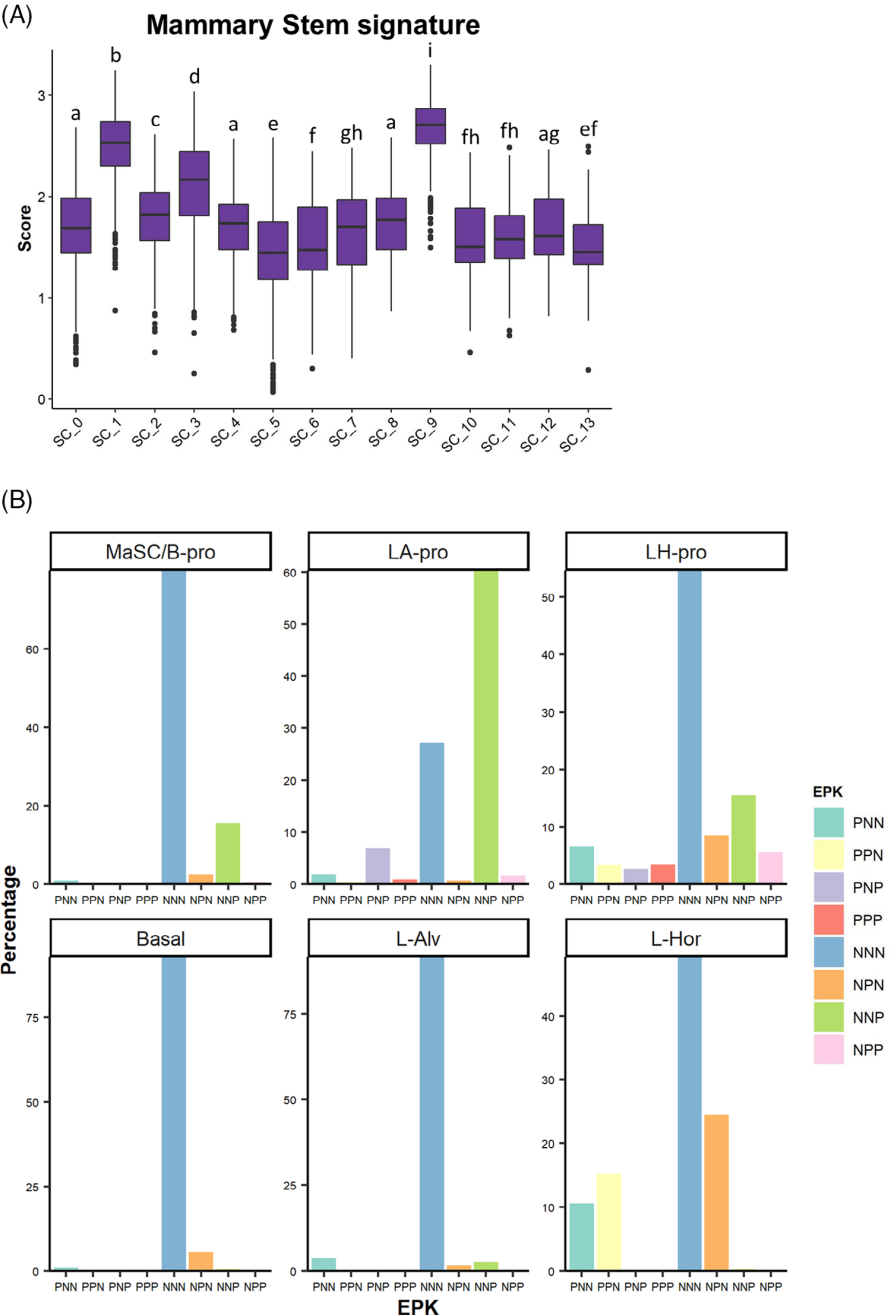
Progenitor‐like features of the *MKI67^+^* proliferative cell subsets. (A) Mammary ‘Stem’ gene signature scoring on the GS3/SC31 scRNA‐seq dataset. The gene list for the mammary stem signature was derived from our previous literature^23^ and the score was calculated using the VISION R package. Different letters on the box plots indicate significant difference between the groups (*p* < .05). (B) EPK classification of normal mammary epithelium dataset. Each panel shows the results on each lineage of cells identified in our previous study.^23^

Our analysis of ER^+^ PDXs, including ST and scRNA‐seq analyses, indicated that the *MKI67* ^+^ ST_2 compartment was the major driver for the proliferation of ER^+^ cancers. We previously established a normal mammary epithelial cell atlas and curated gene signatures representing distinct epithelial lineages.^23^ Our analyses have suggested that luminal A cancers have their origins in the ‘mature’ L‐Hor cell lineage, and luminal B type is more associated with the progenitor state (LH‐pro).^23^ Remarkably, in the current study, the proliferative SC clusters (SC_1, SC_3 and SC_9) had higher scores for the mammary ‘Stem’ signature developed from the epithelial atlas (Figure 8A), suggesting similarities between these *MKI67* ^+^ cells and the normal mammary stem/progenitor‐like cells. In the normal mammary epithelial cell atlas, we also found that the *Mki67* ^+^ cells were only observed in the progenitor populations (Figure 8B). NNP cells were found in all three progenitor populations, whereas PNP, NPP and PPP cells were only found in the LA‐pro and LH‐pro populations. NPP and PPP cells were more abundant in the LH‐pro population than in the LA‐pro population, suggesting their more differentiated features toward L‐Hor lineage.

## 4 DISCUSSION

The importance of single‐cell and ST research in clinical and translational medicine has been clearly recognised.^53^ For the first time, we revealed the intratumour heterogeneity within ER^+^ breast cancer by the application of ST and scRNA‐seq analyses on three unique models, including two ‘ER‐high’ PDXs (GS3 and SC31) with opposite responses to oestrogen, followed by validation through an oestrogen‐accelerating ‘ER‐low’ PDX (GS1). From ST analysis, we identified four spatially separated compartments (i.e., oestrogen‐responsive, proliferative, hypoxia‐induced, and inflammation‐related), which showed unique gene signatures as previously reported mechanisms of cancer development and progression.^44^, ^45^, ^46^, ^47^, ^48^, ^49^ Among our key findings, the responsiveness of a highly proliferative cell population with progenitor cell‐like features (i.e., ST_2) to oestrogen was shown to be crucial for ER^+^ cancer growth, leading to the acquisition of luminal B features. In contrast, oestrogen‐responsive cells (in ST_0) may be linked to mature hormone‐sensing lineage. The ‘proliferative’ cell population was further validated using an independent published single cell dataset.^21^ Through the analysis of clinical databases, the four major compartments were shown to be associated with breast cancer patient outcomes in a distinct manner, suggesting the translational implication of our findings from ST analysis.

The association between the oestrogen‐regulated gene expression (e.g., *PGR*, *AREG*) and the oestrogen‐dependent growth has been extensively investigated.^54^, ^55^, ^56^, ^57^, ^58^ Previous studies mainly used breast cancer cell lines, especially MCF‐7, which are known to show concomitant up‐regulation of oestrogen‐responsive genes and cell proliferation genes within the same cell responding to oestrogen. The results in the current study on the tumour models with the cells showing heterogenous gene expression indicated that the expansion of cells expressing typical oestrogen‐regulated genes like *PGR* would not be directly associated with oestrogen‐mediated proliferation. Functionally, the gene expression signatures of two major oestrogen‐regulated cell clusters (i.e., ST_0 and ST_2) were linking to opposite survival outcome (Figure 7). Expression of oestrogen‐regulated genes could indicate a better response to the endocrine therapy, as we observed a better clinical outcome associated with ST_0 gene signature (Figure 7). Our findings support the observation in clinical practice that luminal A (low ST_2 gene signature) patients with higher oestrogen‐regulated gene expressions show better prognoses compared with luminal B (high ST_2 gene signature) patients with low oestrogen response and high proliferation.^2^, ^3^, ^5^ While Scabia et al.^59^ described the inter‐patient tumour heterogeneity of the hormone response, our ST and scRNA‐seq data revealed that the intratumour heterogeneity, with physically separated ‘oestrogen‐responsive’ and ‘oestrogen‐proliferative’ cell compartments, might explain the biological basis of two types of luminal cancers. Although progesterone was indicated to suppress the oestrogen‐dependent proliferation of MCF‐7 cells,^60^ a progesterone treatment did not affect the proliferation nor the bulk gene expression pattern of oestrogen‐suppressed GS3 (Figures S23A and B). Overall, our results highlight the power of ST technology together with biologically defined PDXs for identifying physically separated functional compartments in ER^+^ breast cancer.

The *MKI67* ^+^ cells were exclusively included in the SC clusters correlating with ST_2. Our results supported the previous clinical study that only Ki67, but not ER/PR, was the negative factor for patient prognosis in IHC3, an IHC‐based scoring method.^61^ Among the *MKI67^+^* cells, we identified two distinct classifications of the cells correlated with oestrogen‐dependent growth, that is, PNP and PPP/NPP cells. PNP cells could be observed both with and without E2 and were implied to be associated with oestrogen‐dependent tumour growth in luminal/ER^+^ breast cancers. Meanwhile, our results suggested that PPP/NPP cells were solely oestrogen‐dependent and present in a less aggressive luminal A‐like subtype. Although our approach based on *ESR1*/*PGR*/*MKI67* expressions may include false negative classifications due to ‘drop‐out’ during the ST/scRNA‐seq experiments, the following dual IHC analysis, as well as our previous results, supported the identification of each class of cells and their responses to the treatments. Intriguingly, palbociclib could suppress ER^+^Ki67^+^ (PNP), but not PR^+^Ki67^+^ (PPP/NPP) cells, providing the potential explanation of why palbociclib has been only effective for advanced breast cancers and highlighting the importance of its combination with endocrine therapy to target both PNP and PPP/NNP populations. The additional examination on GS1 model indicated that NNP cells could be the dominant cell type in driving cancer growth in ‘ER‐low’ breast cancers. It is noted that the NNP cell population in GS1 increased after oestrogen treatment as it implied potential interactions between *ESR1* ^+^ and *ESR1* ^−^ proliferative cells. In the normal mammary epithelium dataset, NNP cells were found in the mammary stem or basal progenitor populations, implying that they are present not only in luminal cancers but also in other subtypes such as basal‐like cancers. Collectively, our study would indicate the complexity of cell distribution driving the tumour proliferation in ER^+^ breast cancers, that is, ER‐positive (PNP and PPP/NPP) and ‐negative (NNP) cells, and our findings support the importance of combination therapy to suppress all proliferative populations within a single tumour.

Our ST analysis on our PDX models found a compartment with higher hypoxia‐induced signatures. Hypoxia is one of the hallmarks of cancers and often occurs alongside necrosis formed by insufficient oxygen and nutrition supply. The hypoxia‐induced phenotypical changes of breast cancer cells have been thought to be important especially for distal metastasis.^45^ Indeed, our hypoxia‐induced cluster concomitantly showed the enrichment of gene signatures known to promote metastasis. Notably, the hypoxia‐related gene signature in ST_5 was not affected much by oestrogen treatment in this study. Also, the non‐luminal breast cancers in the METABRIC cohort showed higher expressions of the ST_5 gene signature, indicating the importance of hypoxic features as oestrogen‐independent mechanisms of tumour progression for all breast cancer subtypes. Our ST analysis has revealed that glycolysis is linked to ST_5, while oxidative phosphorylation is associated with proliferative ST_2 that requires efficient energy production (Figure 3C). These results pose the opportunity for future combination treatment of endocrine therapy with hypoxia‐targeting agents (e.g., anti‐angiogenic therapy) to suppress metastasis.

Our ST analysis identified another compartment showing the inflammation‐related signatures, especially with the up‐regulation of IFN‐responsive genes. IFNs have been investigated as inflammatory mediators produced by immune cells, facilitating anti‐tumour immunity. However, emerging evidence suggests that the tumour cells themselves can produce IFNs and thereby activate IFN signalling autonomously.^62^ A recent study demonstrated that the potential autonomous activation of IFN signalling in ER^+^ breast cancers led to the CDK4/6 inhibitor resistance.^48^ Also, the long‐term inhibition of oestrogen signalling in breast cancer cells can induce IFN‐responsive gene expressions, leading to endocrine resistance.^47^, ^63^ In our study, we observed the downregulation of IFN‐response signatures in ST_7 with E2 treatment. Considering that our SC31 model was partly suppressed by endocrine therapy in our previous study,^15^ we speculate that the IFN‐responsive signalling would be usually suppressed by oestrogen in ER^+^ breast cancers, but once the oestrogen signalling was inhibited, the IFN‐responsive population would become dominant for the breast cancer's progression. While further mechanistic studies are needed, this study identifies additional therapeutic target that could be used to improve clinical outcomes in ER^+^ breast cancers.

The design of our study required well‐defined oestrogen‐responsive models. Importantly, we have PDX models with different responses to oestrogen treatment. A limitation of this study is that we mainly focused on two ER^+^ breast cancer PDXs, GS3 and SC31, with confirmative evidence from another ‘ER‐low’ PDX GS1. Yet, consistent intratumour heterogeneity was clearly demonstrated in these structurally and functionally distinct tumour models. While the translational value of scRNA‐seq analysis on clinical specimens can be significantly compromised because of their uncontrolled quality, the recent paper by Wu et al.^21^ performed large‐scale scRNA‐seq analyses on clinical samples and determined seven cell groups with distinct gene signatures called ‘gene module (GM)’ across all breast cancer subtypes. Importantly, their GMs corresponded to our ST/SC signatures (e.g., the proliferation‐related gene signatures in GM3 or the IFN‐responsive genes in GM4). Our analysis on the nine ER^+^ specimens from their dataset confirmed the presence of the proliferative cell population (i.e., cluster 9 in Figure 5G), well separated from other cell populations which were compromised by the heterogeneous number of cells from different patients.

Our comprehensive ST analyses on ER^+^ PDXs with defined oestrogen responses, for the first time, revealed functionally and physically distinct compartments within ER^+^ breast tumours and demonstrated the importance of the ST_2 signature, including Ki67 expression, using the METABRIC and two other cohorts. Again, the strength of our study using PDXs is the ability to evaluate the response to experimental intervention, that is, oestrogen treatment. While the use of PDXs limits the investigation of impact of tumour microenvironment, the unique approach on the two PDXs with opposite responses to oestrogen allowed us to elucidate the essential cell population for oestrogen‐dependent tumour growth. While multiple important analyses on correlations between bulk gene profiling and clinical outcome have been carried out using primary tumour specimens, a limitation is the lack of direct evidence for relationship between gene expression and tumour growth. Thus, our findings are crucial not only for improving the understanding of ER^+^ breast cancer biology, but also for providing insights to develop more targeted therapeutic strategies toward better patient prognoses.

## 5 CONCLUSIONS

**FIGURE 9:**
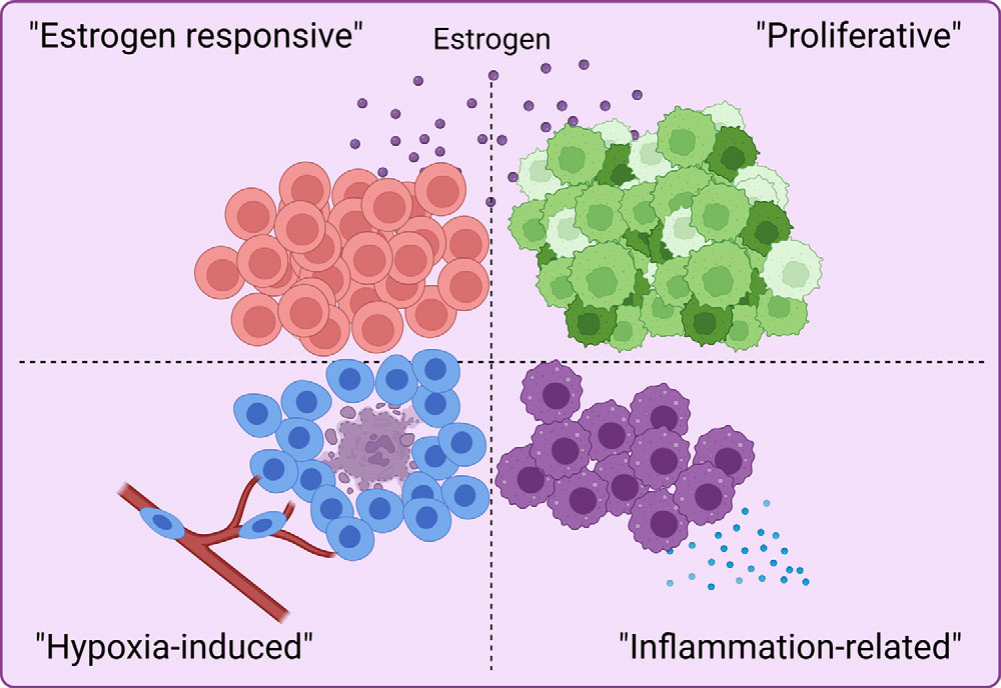
Graphical abstract. We identified four spatially separated populations in ER^+^ breast cancers with distinct gene signatures, leading to overall cancer progression in a function‐specific manner.

Our space‐gene‐function study identified the four active populations in ER^+^ breast cancers (i.e., oestrogen responsive, proliferative, hypoxia‐induced and inflammation‐related) (Figure 9). These spatially distinct functional compartments were suggested to uniquely contribute to breast cancer progression. We identified a cell cluster linked to oestrogen‐dependent tumour proliferation that were different, spatially and functionally, from cells which express typical oestrogen‐responsive genes. Moreover, the proliferative cells were further distinguished into more specific cell subsets, with different cell cycle phases, in the scRNA‐seq analysis, implicating their distinct contribution toward tumour growth. Overall, our study on oestrogen‐responsive PDXs through ST and scRNA‐seq analyses provides a comprehensive view of intratumour heterogeneity in ER^+^ breast cancers and reveals a proliferative compartment as the biological and mechanistic basis of luminal B breast cancer.

## AUTHOR CONTRIBUTIONS

R. Y. and S. C. designed the study. R. Y., H. M., H. J. S. and Y. C. performed animal experiments. D. H. and X. Wang performed histological analyses. X. Wu and J. W. performed the sequencing. R. Y., X. Wu, K. S. and G. C. performed bioinformatic analyses. S. C. supervised the study. R. Y., H. M., D. H., X. Wang, K. S., G. C., Y. C. and S. C. drafted and revised the manuscript. All authors read and approved the final manuscript.

## CONFLICT OF INTEREST STATEMENT

The authors declare that there is no conflict of interest.

## ETHICS STATEMENT AND CONSENT TO PARTICIPATE

Animal experiments performed in this study were approved by the Institutional Animal Care and Use Committee at City of Hope and were operated according to the institutional and National Institutes of Health guidelines for animal care and use.

## CONSENT FOR PUBLICATION

Not applicable.

## Supporting information

Supporting Information

Supporting Information

Supporting Information

## Data Availability

The ST datasets obtained in this study (GS3‐Placebo, GS3‐E2, SC31‐Placebo and SC31‐E2) and the scRNA‐seq datasets from GS1 are openly available in the National Center for Biotechnology Information Gene Expression Omnibus data repository at https://www.ncbi.nlm.nih.gov/geo/, accession number GSE214571 and GSE213733, respectively. The codes and data files generated in this study are available on Zenodo (https://doi.org/10.5281/zenodo.10435306).
