# Supplementary material for: Molecular features of luminal breast cancer defined through spatial and single‐cell transcriptomics
Source: Clin Transl Med. 2024 Jan 28;14(1):e1548. doi: 10.1002/ctm2.1548 (PMC10823285; doi:10.1002/ctm2.1548)
Supplement: Supplementary file 1 — Supporting Information [file CTM2-14-e1548-s001.docx]

**Supplementary Figure legends**

**Figure S1. Expression of *ESR1*/*PGR*/*MKI67* genes in the ST datasets.**

The ridge plot shows the distribution of levels of gene expressions in each tissue. X axis represents the log-normalized counts. Dash line indicates the cutoff value of 0.2.

**Figure S2. H&E staining and IHC for ER/PR/Ki67/HER2 in GS1 model.**

Scale bar = 100 µm.

**Figure S3. Growth responses of ER^+^ breast cancer PDX models to estrogen treatment.**

**A** Growth curves of the E2-accelerating (GS1, GS2) and -dependent (GS4) breast cancer PDXs. Data are shown as mean ± SEM. *, *P* < 0.05; **, *P* < 0.01. **B** Tumor volume changes of GS3 and SC31 samples used for the ST experiment.

**Figure S4. H&E-stained images of the sections used for the ST experiment.**

* represents scar-like tissue area observed in SC31 tissues. Scale bar = 2 mm.

**Figure S5. QC metrics of the datasets obtained from the ST experiment.**

The spatial plot on the left shows the location and level of the QC metric values on each tissue. The violin plot on the right summarizes the QC metrics in each tissue.

**Figure S6. Expression of mouse-derived stromal cell markers in the ST datasets.**

The spatial plot on the left shows the location and level of the expression on each tissue. The violin plot on the right summarizes the gene expressions in each tissue.

**Figure S7. Expression of human-derived stromal cell markers in the ST datasets.**

The spatial plot on the left shows the location and level of the expression on each tissue. The violin plot on the right summarizes the gene expressions in each tissue.

**Figure S8. Comparison of human- and mouse-derived gene expression in the ST datasets.**

The proportion of human- and mouse-derived genes in each spot. Red and blue spot in the bottom panels represents the spot including more human and mouse genes, respectively.

**Figure S9. Cell cycle phases of the spots in the ST datasets.**

**Figure S10. Expression of *IL24* in the ST datasets.**

The spatial plot on the left shows the location and level of the expression on each tissue. The violin plot on the right summarizes the gene expressions in each tissue. *, *P* < 0.05; ns, not significant.

**Figure S11. Clustering on the integrated ST dataset.**

**A** UMAP plot of the integrated dataset. Spot is represented by the dot and is colored according to the cluster (ST_0–8) identified by the unbiased clustering (left) or the originated datasets (right). **B** QC metrics of the ST clusters.

**Figure S12. Localization of the ST clusters on each tissue.**

Yellow dot shows the localization of the ST cluster (ST_0-8) indicated on the left.

**Figure S13. Estrogen-response gene signature scores in the ST clusters.**

The score was calculated using VISION R package. Different letters on the box plots indicates significant difference between the groups (*P* < 0.05).

**Figure S14. Cell cycle phases of the spots included in each ST cluster.**

**Figure S15. Analyses on the integrated scRNA-seq dataset from GS3 and SC31.**

**A** QC metrics of the clusters (SC_0-13) identified in the scRNA-seq dataset. **B** Heatmap of the top 10 genes for the SC clusters. Column represents the cells and row represents the genes. The gene expression levels were scaled by SCTransform function. **C** Comparison of expression patterns of the cluster-specific genes between the ST and scRNA-seq datasets.

**Figure S16. Expression of *MKI67* gene among the SC clusters.**

The violin plot summarizes the gene expressions in each SC cluster.

**Figure S17. AUCell scores for the spatial mapping among the ST clusters.**

The score was calculated using AUCell R package. Different letters on the box plots indicates significant difference between the groups (*P* < 0.05).

**Figure S18. Expression of *ESR1*/*PGR*/*MKI67* genes in the integrated scRNA-seq dataset from GS3 and SC31.**

The violin plot summarizes the gene expressions in each sample. *, *P* < 0.05; ns, not significant.

**Figure S19. Analyses on the integrated scRNA-seq dataset from human ER^+^ breast cancers.**

**A** QC metrics of the individual datasets in the integrated human scRNA-seq dataset (n = 9). **B** The number of normal or cancer epithelial cells from the individual datasets. **C** Heatmap of the top 10 genes for the clusters. Column represents the cells and row represents the genes. The gene expression levels were scaled by SCTransform function.

**Figure S20. Quantification of the dual IHC for ER/Ki67 and Ki67/PR.**

The dual IHC results for **A** ER/Ki67 and **B** Ki67/PR. Bar plot indicates the number of the cells classified as indicated per field. Data are shown as mean ± SEM [n = 4 (E2) and 5 (E2 + Palbo)]. *, *P* < 0.05; **, *P* < 0.01; ns, not significant.

**Figure S21. Clinical data analysis with ST_0 and ST_2 signatures on GSE124647 cohort.**

Stage IV ER^+^/HER2^-^ breast cancers in the GSE124647 cohort (n = 140) were analyzed using ST_0 and ST_2 signatures. The gene signature scores were calculated in each patient using GSVA R package. Kaplan-Meier plots show the overall survival of patients in each group with *P* values at the bottom left corner.

**Figure S22. Clinical data analysis with the ST signatures on all breast cancer subtypes in METABRIC cohort.**

All breast cancers in the METABRIC cohort (basal-like, n = 199; claudin-low, n = 199; HER2-enriched, n = 220; luminal A, n = 679; luminal B, n = 461) were analyzed using the gene signatures from ST_0, ST_2, ST_5, and ST_7. The gene signature scores were calculated in each patient using GSVA R package. Left panels show box plots of the scores in each subtype. Different letters on the box plots indicate significant difference between the subtypes (*P* < 0.05). Right panels show Kaplan-Meier plots for the overall survival of the patients in each group with *P* values at the bottom left corner.

**Figure S23. Effect of progesterone treatment on GS3 model.**

**A** Growth curve of GS3 in control, E2, P4, and E2 + P4 groups. Data are shown as mean ± SEM. Different letters indicate significant difference between the groups on day 28 (*P* < 0.05). **B** Heatmap of the results from bulk RNA-seq analysis of GS3 in control, E2, P4, and E2 + P4 groups. Row represents the samples and column represents the genes. The expression levels of the genes were scaled per column.
